# An investigation of depression, anxiety, and stress and its relating factors during COVID-19 pandemic in Iran

**DOI:** 10.1186/s12889-021-10329-3

**Published:** 2021-02-03

**Authors:** Fatemeh Khademian, Sajad Delavari, Zahra Koohjani, Zahra Khademian

**Affiliations:** 1 Department of Health Information Management, School of Management and Medical Information Sciences, Shiraz University of Medical Sciences, Shiraz, Iran; 2 Health Human Resources Research Center, School of Management and Medical Information Sciences, Shiraz University of Medical Sciences, Shiraz, Iran; 3 Community-Based Psychiatric Care Research Center, Department of Nursing, School of Nursing and Midwifery, Shiraz University of Medical Sciences, Shiraz, Iran

**Keywords:** Stress, Depression, Anxiety, COVID-19, Coronovirus, SARS-CoV-2, Mental health

## Abstract

**Background:**

The COVID-19 (SARS-CoV-2) is an emerging epidemic caused by the new Coronavirus. It has affected more than 200 countries, infected 5,939,234 people, and killed 367,255 in the world until 1 June 2020. While the disease epidemic could affect population mental health, this study aimed to investigate stress, anxiety, and depression during the Corona pandemic in Iran.

**Methods:**

An online survey was designed using the depression, anxiety, and stress scale (DASS-21) questionnaire. The questionnaire was available for all Iranian population from 18 to 28 April 2020. Finally, 1498 participants filled the questionnaire using snowball sampling. Data were analyzed using multivariate regression models.

**Results:**

Findings showed that most participants had experienced a normal level of stress (36.6%), anxiety (57.9%) and depression (47.9%). About 2.5% of respondents report an extremely severe level of stress. This amount of anxiety and depression was 6.3 and 7.9%, respectively. Regression model showed being female (CI: − 1.299; − 0.248), living with a high risk family member (CI: 0.325; 1.400), health status (CI: − 0.857; − 0.595), economic status (CI: − 0.396; − 0.141), social capital (CI: − 0.475; − 0.244), risk of disease (CI: 0.081; 0.729), and following COVID-19 news (CI: 0.111; 0.551) have a relation with stress level. Education level (CI: − 0.252; − 0.017), living with a high risk family member (CI: 0.0301; 1.160), health status (CI: − 0.682; − 0.471), social capital (CI: − 0.236; − 0.048), risk of disease (CI: 0.154; 0.674), and following COVID-19 news (CI: 0.046; 0.401) have a relation with anxiety score. Depression score was in relation with education level (CI: − 0.263; − 0.022), having a high-risk family member (CI: 0.292; 1.155), health status (CI: − 0.687; − 0.476), social capital (CI: − 0.235; − 0.048), risk of disease (CI: 0.144; 0.667), and following Covid-19 news (CI: 0.053; 0.408).

**Conclusions:**

Most of the factors related to depression, anxiety, and stress are related to COVID-19, such as having a vulnerable person in the family, risk of disease, and following COVID-19 news. The findings suggest the factors that should be taken into consideration for improving population mental health during pandemics.

## Background

The COVID-19 is an emerging epidemic caused by the Coronavirus (SARS-CoV-2). This disease first occurred in Wuhan, Hubei Province, China, in December 2019 [1], and since then, it has affected more than 200 countries and territories, infected 5,939,234 people, and killed 367,255 in the world until 1 June 2020 [2]. The World Health Organization (WHO), on 11 March 2020, considered COVID-19 as a pandemic [3]. The first peak of this epidemic in Iran occurred in the last days of March 2020. Around this time 3186 new cases were reported in 1 day [4].

According to the Iranian Ministry of Health, the first confirmed case of COVID-19 was reported on 18 February 2020 and has infected 151,466 and killed 7797 Iranians to 22 May 2020 [4]. School classes were also suspended from 29 February 2020. The government tries to set stricter rules to control its outbreak since 27 March 2020, and entry to and exit from cities have been restricted [5]. Besides, some people lost some or all parts of their income during this outbreak [6].

According to current epidemiological research, the incubation period of Covid-19 ranged from one to fourteen days. The most common symptoms in patients include fever, dry cough, and shortness of breath [7]. People with underlying diseases such as diabetes, cardiovascular disease, renal failure, obesity, and immunodeficiency are believed to be more vulnerable [8].

During pandemics or epidemics, there are mental and social disorders that could disrupt population activity. Fear of getting sick makes the condition worse. During pandemics, the community experiences stress and anxiety to some extent, and psychological disorders become common [8]. It is estimated that between one-third to one-half of people may experience psychiatric problems in these situations [9]. Extensive infectious diseases usually affect human survival. Since most people do not know about emerging infections in the first stages, they are more affected by social and psychological problems, which generally cause widespread fear and irrational reactions to it [10]. An increase in the number of deaths following the epidemic leads to worries in communities due to misinformation, stress, sadness, pervade societies, and emotional confusion, lead to out-of-control behaviors. In this situation, policymakers need to take appropriate actions to improve individuals’ mental health [11–13]. Therefore, there is a need for more studies on the general population’s mental health aspects during the COVID-19 pandemic.

Previous research performed during the initial phase of the coronavirus outbreak has revealed more than half of the respondents rated the psychological impact as moderate-to-severe, and about one-third reported moderate-to-severe anxiety [14]. During the Severe Acute Respiratory Syndrome (SARS) outbreak in 2003, several studies were performed in some countries, mostly in Hong Kong [15]. For example, researchers showed higher anxiety levels among healthcare workers who had contact with SARS patients [16]. Those at increased risk of SARS infection also appeared to have chronic stress and higher depression and anxiety [17]. Individuals who had a heightened sense of risk perception and moderate anxiety levels were more likely than others to take personal protective measures against infection [18]. Moreover, a study aimed to investigate the psychological impact of the pandemic H1N1 (2009) on hospital workers and found that workers that had a high risk of infection felt more anxious and feel more tiredness at workplace environments [19]. At the early outbreak phase of the H1N1, Lau et al. concluded that the general population exhibited avoidance behaviors and negative psychological responses [20].

Given the current worldwide concern over the spread of the COVID-19, people are faced with several limitations, affecting their mental health. The Iranian population has not experienced such restrictions for disease in recent decades. Therefore, the study on population mental health in pandemics such as COVID-19 is new, and there is limited knowledge about DAS^1^ status during the COVID-19 pandemic. This study aimed to investigate the depression, anxiety, and stress among the Iranians during the COVID-19 outbreak. The findings will improve our understanding of the psychological impact of exposure to an outbreak of a fast-spreading, life-threatening infectious disease and strengthen preparations for responding to possible future outbreaks or pandemics of contagious diseases.

## Methods

This online survey was conducted using an anonymous online questionnaire in the Persian language from 18 to 28 April 2020. We utilized a snowball sampling strategy focused on recruiting the general population living in Iran during the epidemic of COVID-19. The online survey was disseminated to people in different provinces of Iran. For sample selection, we divided the country into four regions based on ethnicity and population distribution. We expected to select a sample of 384 participants from each region based on the Krejecie and Morgan sample size table [21]. The total desired sample size was 1536. The questionnaire link was disseminated in Iran’s favorite social networks, including Whatsapp and Instagram. The respondents were encouraged to pass it on to others. Participants completed the questionnaire individually in an estimated mean time of 7 min through an online survey platform. We complied with ethical research standards by providing information on the project and requesting consent to participate in all cases. So, all respondents provided informed consent.

The structured questionnaire consisted of questions that covered two sections: 1) socio-demographic data, 2) self-reported stress, anxiety, and depression during the COVID-19 pandemic. Socio-demographic data included gender, age, marital status, degree, urbanity, change in income during the COVID-19 outbreak, exposure to an infected case, exposure to people with Covid-19 symptoms, and having a high-risk family member. Respondents were asked to fill out the questionnaire according to their experience in the past month. Since the questionnaire was distributed online and self-reported, participants were provided with two cellphone numbers at the questionnaire introduction section to ask any questions that may arise while filling the questionnaire. In about 2% of responses, respondents made phone calls to ask their questions.

Stress, anxiety, and depression were assessed using the Depression, Anxiety, and Stress Scale (DASS-21). Depression subscales included seven questions. The total depression subscale score was considered normal (0–9), mild (10–12), moderate (13–20), severe (21–27), and extremely severe (28–42). Anxiety subscales included seven questions. The total anxiety subscale score was considered normal (0–6), mild (7–9), moderate (10–14), severe (15–19), and extremely severe (20–42). Stress subscales included seven questions. The total stress subscale score was considered normal (0–10), mild (11–18), moderate (19–26), severe (27–34), and extremely severe (35–42). The test interpretation was done under the questionnaire guideline with the help of a clinical psychology expert.

The DASS questionnaire has been a valid and reliable tool for assessing depression, anxiety, and stress levels among the Iranian population [22]. Cronbach alpha for the Persian version of the questionnaire was reported 0.85, 0.85, and 0.87 for depression, anxiety, and stress, respectively [23]. In another study, the Cronbach alpha for all items was 0.89 [24]. These values indicate the Persian version of DASS-21 has acceptable internal consistency. Asghari et al. checked the reliability using test-retest with a three-week interval on 40 participants. The Pearson coefficient for depression anxiety, and stress was reported 0.77 (95% CI: 0.56–0.88), 0.89 (95% CI: 0.81–0.94) and 0.85 (95% CI: 051–0.94), respectively. From 0.84 (for depression) to 0.90 (for stress) [23]. As well, Asghari et al. reported a strong and positive correlation between three scales of the Persian version of DASS-21 items and the validated versions of Beck’s depression inventory (BDI) and four system anxiety questionnaire (FSAQ) [23]. Therefore, the Persian version of DASS-21 has enough reliability and validity for the Iranian population.

### Data analysis

Data were first analyzed using compare mean tests, including one-sample t-test and ANOVA, and correlation separately for every three outcomes (stress, anxiety, and depression), separately. Gender, marital status, urbanity, exposure to an infected case of COVID-19, exposure to people with Covid-19 symptoms, and having a high-risk family member had two values tested using a one-sample t-test. Education degree and change in income due to covid-19 were considered as multi-value variables and tested using ANOVA. Other variables, including age, perceived health status, perceived economic status, perceived social capital, perceived risk of COVID-19, and following Covid-19 news, were quantitative and had a normal distribution tested using Pearson correlation. The variables, which have a *p*-value less than 0.2 in bivariate analysis, were inserted in three regression models to test their relationship with stress, anxiety, and depression as dependent variables. The statistical significance level was considered 0.05. Data analysis was performed using SPSS and STATA software.

## Results

Totally 1498 individuals filled the questionnaire. As results showed in Table 1, most of the participants have experienced normal and mild stress levels. But 8% of participants have severe or extremely severe stress. While 57.9% of participants have a normal anxiety level, 12.4% have anxiety higher than moderate. As well, about 15% of participants have severe or extremely severe depression (Table 1).

**Table 1:** Stress, Anxiety, and Depression levels of the participants

Variables	Stress	Anxiety	Depression
Normal			
Frequency	549	867	717
Percent	36.6	57.9	47.9
Mild			
Frequency	580	153	262
Percent	38.7	10.2	17.5
Moderate			
Frequency	249	291	298
Percent	16.6	19.4	19.9
Severe			
Frequency	83	92	103
Percent	5.5	6.1	6.9
Extremely severe			
Frequency	37	95	118
Percent	2.5	6.3	7.9

Using the t-test and ANOVA compare mean test, we found stress, anxiety, and depression level vary in different groups (Table 2). The mean stress score for female participants was 14.3, which is significantly higher than that of men (13.6) (*p*-value = 0.008). Participants with post-graduate education had significantly lower stress levels, anxiety, and depression than other educational groups. Rural participants have a higher level of depression than urban ones (*p*-value = 0.038). People who lost some or all of their income during the COVID-19 outbreak experienced a higher level of stress (*p-*value = 0.001) and depression (*p-*value< 0.001) than other people. The people who live with a high-risk family member to COVID-19 experienced a significantly higher degree of stress, anxiety, and depression. The score was 14.5, 10.8, and 13 for stress, anxiety, and depression, respectively. These scores were 13.1, 9.8, and 11.8 for those who do not live with a high-risk person. Level of stress, anxiety, and depression did not vary significantly based on marital status, exposure to infected cases of COVID-19, and exposure to people with COVID-19 symptoms.

**Table 2:** Frequency of participants based on demographic variables and results of compare means test

variable	Division (Number)	Stress Score		Anxiety Score		Depression Score	
		Mean ± SD	*p*-value	Mean ± SD	*p*-value	Mean ± SD	*p*-value
Gender	Male (340)	13.6 ± 4.23	0.008	10.5 ± 3.50	0.458	12.6 ± 4.71	0.871
	Female (1158)	14.3 ± 4.20		10.6 ± 3.33		12.7 ± 4.54	
Marital status	Single (675)	14.0 ± 4.17	0.256	10.5 ± 3.31	0.117	12.9 ± 4.61	0.065
	Married (823)	14.2 ± 4.25		10.7 ± 3.42		12.5 ± 4.51	
Education level	Diploma and below (339)	14.3 ± 4.27	0.001	10.9 ± 3.57	0.001	13.0 ± 4.80	0.013
	Undergraduate (698)	14.4 ± 4.23		10.8 ± 3.42		12.9 ± 4.60	
	Postgraduate (461)	13.5 ± 4.10		10.1 ± 3.08		12.2 ± 4.32	
Urbanity	Urban (1368)	14.1 ± 4.19	0.344	10.6 ± 3.33	0.590	12.6 ± 4.53	0.238
	Rural (130)	14.5 ± 4.51		10.8 ± 3.70		12.9 ± 4.99	
Change in Income during Covid-19 outbreak	Without job (464)	14.3 ± 4.36	0.001	10.5 ± 3.29	0.234	12.9 ± 4.85	> 0.001
	Decreased (389)	14.6 ± 4.16		10.8 ± 3.45		13.1 ± 4.60	
	Without change (616)	13.8 ± 4.11		10.6 ± 3.38		12.3 ± 4.35	
	Increased (29)	12.1 ± 3.89		9.7 ± 3.24		10.1 ± 3.17	
Exposure to infected case	Yes (296)	14.5 ± 4.17	0.133	10.9 ± 3.53	0.073	13.0 ± 4.61	0.332
	No (931)	14.1 ± 4.30		10.5 ± 3.28		12.7 ± 4.65	
Exposure to people with Covid-19 symptoms	Yes (141)	14.7 ± 4.27	0.157	10.9 ± 3.65	0.219	13.2 ± 4.77	0.268
	No (1021)	14.1 ± 4.27		10.5 ± 3.31		12.7 ± 4.62	
Having a high-risk family member	Yes (897)	14.5 ± 4.23	< 0.001	10.8 ± 3.44	> 0.001	13.0 ± 4.67	< 0.001
	No (266)	13.1 ± 4.24		9.8 ± 2.94		11.8 ± 4.41	

Quantitative demographic variables related to stress, anxiety, and depression were tested using correlation tests, shown in Table 3. As the results revealed, age was not significantly associated with stress, anxiety, and depression. But, perceived health status, perceived economic status, and perceived social capital had a negative and significant association with stress, anxiety, and depression. The perceived risk of COVID-19 and following COVID-19 news had a positive and significant relationship with the main study variables.

**Table 3:** Correlation between demographic variables and stress, anxiety and depression scores

Variables	Stress score		Anxiety score		Depression score	
	coefficient	*p*-value	coefficient	*p*-value	coefficient	*p*-value
age	−0.024	0.347	0.028	0.279	− 025	0.326
Perceived health status	−0.394	< 0.001	−0.380	< 0.001	− 0.367	< 0.001
Perceived economic status	−0.244	< 0.001	− 0.154	< 0.001	−0.265	< 0.001
Perceived social capital	−0.269	< 0.001	−0.182	< 0.001	− 0.277	< 0.001
Perceived risk of disease	0.150	< 0.001	0.148	< 0.001	0.101	< 0.001
Following Covid-19 news	0.121	< 0.001	0.120	< 0.001	0.096	< 0.001

The variables with a *p-*value of less than 0.2 were inserted in linear regression models to find their effect on study variables. Being female, having a high-risk family member, having lower health status, having a lower economic level, having lower social capital, considering covid-19 as a dangerous disease, and following COVID-19 news significantly was related to stress score. Anxiety score was significantly higher among less-educated participants and those with a high-risk family member, lower health and economic status, and lower social capital. As well, the participants who consider the COVID-19 more dangerous and follow its news have a significantly higher level of anxiety. Having a high-risk family member, lower health status, lower social capital, considered higher risk for COVID-19, and more checking of COVID-19 news were significantly related to the level of depression (Table 4).

**Table 4:** Regression model on factors affecting stress, anxiety, and depression

variables	Stress Score		Anxiety Score		Depression Score	
	Coef.	95% CI	Coef.	95% CI	Coef.	95% CI
Education level	0.085	−0.104; 0.274	− 0.135	− 0.252; − 0.017	− 0.143	− 0.263; − 0.022
Gender (male)	−0.773	−1.299; − 0.248	NI	NI	NI	NI
Marital status (single)	NI	NI	0.102	−0.258; 0.461	0.089	−0.274; 0.452
Change in income (without a job)			NI	NI		
Decreased	0.072	−0.519; 0.664			0.121	−0.359; 0.600
Without change	−0.309	− 0.854; 0.235			0.146	−0.295; 0.587
Increased	−0.525	−2.196; 1.146			−0.003	−1.346; 1.340
Exposure to infected case	0.023	−0.505; 0.550	0.155	−0.263; 0.573	NI	NI
Exposure to people with Covid-19 symptom	0.217	−0.478; 0.913	NI	NI	NI	NI
Having a high-risk family member	0.863	0.325; 1.400	0.730	0.0301; 1.160	0.723	0.292; 1.155
Perceived health status	−0.726	−0.857; − 0.595	−0.577	− 0.682; − 0.471	−0.581	− 0.687; − 0.476
Perceived economic status	−0.268	− 0.396; − 0.141	−0.090	− 0.190; 0.011	−0.093	− 0.197; 0.011
Perceived social capital	−0.359	− 0.475; − 0.244	−0.142	− 0.236; − 0.048	−0.141	− 0.235; − 0.048
Perceived risk of disease	0.405	0.081; 0.729	0.413	0.154; 0.674	0.406	0.144; 0.667
Following Covid-19 news	0.331	0.111; 0.551	0.224	0.046; 0.401	0.230	0.053; 0.408
Constant	19.228	16.888; 21;568	12.521	10.717; 14.325	12.988	10.975; 15.002
Prob > F	< 0.0001		< 0.0001		< 0.0001	
R-squared	0.2306		0.1761		0.1765	
Adj R-squared	0.2219		0.1696		0.1678	

*Coef.* Coefficient, *CI* Confidence Interval, *NI* Not Inserted in the regression model

## Discussion

Our primary goal in this study was to examine the levels of depression, anxiety, and stress in the Iranian population during the Covid-19 epidemic. Our findings showed that being female, living with a high-risk family member to COVID-19, perceived risk of COVID-19, and following COVID-19 news were associated with a greater level of depression, anxiety, and stress.

Our findings revealed that the symptoms of stress, anxiety and depression were seen in 63.4, 42.1, and 52.1% of the population, respectively. This study is similar to other studies conducted earlier in Iran using the DASS-21 questionnaire. A previous study in Iran showed that stress, anxiety, and depression were reported in 34.8, 32.2, and 29% of adult residents of Yazd Greater Area, respectively [25]. Moreover, another study revealed that 41.8% of elderly residents of Khoy, Iran had stress [26]. Furthermore, a study conducted on the Iranian students in Mashhad showed that depression, anxiety, and stress were reported in 43.3, 43.3, and 38.9 of them [27]. Given these findings, it can be concluded that the level of stress, anxiety and depression might be increased among the Iranian population during the COVID-19 outbreak.

Our results showed that Iranian females experienced a higher level of stress. This finding is consistent with studies in China and Kurdistan of Iraq, which found that women suffered a higher level of depression, anxiety, and stress during the Covid-19 outbreak [14, 28]. However, several studies revealed women experience a higher stress level than men in many situations [29, 30]. Therefore, it may be concluded that their stress may not be affected just by the COVID-19 pandemic situations. Besides, participants with post-graduate education have significantly lower levels of DAS than other educational groups. In this regard, a study in China showed that the general public with no formal education had a higher chance of depression during the Covid-19 epidemic [14]. These results indicate that people with higher education experience less stress and vice versa. Therefore, during outbreaks, more attention needs to be paied to rural populations and less educated people and provide them with the necessary information in a simple and understandable language to increase their knowledge and reduce stress.

Our results showed that stress and depression were directly associated with the financial consequences of the COVID-19. People who lost some or all of their income during the COVID-19 outbreak experienced a higher level of stress and depression than other people. A similar study by the Pew Research Center in the United States found that people with lower incomes and people whose jobs or revenues decreased during the Covid-19 outbreak were more likely to have high distress levels than other groups [31]. So governments should provide financial support and adopt appropriate economic policies to alleviate people’s concerns.

We also found that people who live with a high-risk family member to COVID-19 significantly experienced a higher DAS degree. Consistent with these results, a study showed that 75.2% of Chinese people were anxious about other family members getting COVID-19 [14]. This stress may arise from close family relationships and concerns about family members’ health conditions among Eastern communities. Interestingly, exposure to people with COVID-19 symptoms was not significantly associated with stress, anxiety, and depression, but having a high-risk family member led to an increase in the level of three variables. Therefore, mental health professionals should pay attention to the role of family members’ health in people’s mental health and pay enough attention to this in their support programs.

Our results indicate that following COVID-19 news positively relates to depression, anexiety, and stress levels. It can result from incorrect statistical information and rumors distribute in cyberspace. In this regard, the government and health policymakers must provide accurate and updated information to prevent false information and public health statistics. In this regard, a study showed that up-to-date and accurate health information is associated with a lower level of stress [14]. But it is essential to empower users to be able to use electronic information effectively. In this regard, a study showed that more than 50% of participants had a low level of eHealth Literacy [32].

In our survey, age and marital status did not have significant associations with DAS. Similarly, a study reported that age and marital status were not associated with DAS levels [14]. On the other hand, some studies revealed that younger people in the 21–40 age group were more stressed. They concluded that this group is more stressful than others; because they were active workers and were more affected by the economic crisis due to the COVID-19 outbreak [33].

We found that the perceived risk of COVID-19 has a positive and significant relationship with DAS levels. Similar to our results, a survey reported that low perceived likelihood of contracting COVID-19 was significantly associated with low stress and anxiety [14]. Another study also revealed the perceived risk of COVID-19 infection significantly increased the probability of anxiety or depression [33].

One of this study’s strengths is selecting samples across the country that help in generalizability of the finding to the whole Iranian population. Also, our study provides valuable information from approximately all provinces of Iran. So, our results could be used to make decisions at the national level to reduce mental health problems. On the other hand, because the country was affected by the COVID-19 outbreak, and people were not physically available, we failed to use a random sampling method. So we adopted the snowball sampling method.

## Conclusion

In this study, we identified vulnerable groups to DAS during the COVID-19 pandemic in Iran. We found that being female, living with a high-risk family member, health status, economic status, social capital, risk of disease, and following COVID-19 news is related to stress. Education level, living with a high-risk family member, health status, social capital, risk of infection, and following COVID-19 news is related to anxiety score. Depression score is associated with education level, having a high-risk family member, health status, social capital, risk of disease, and following Covid-19 news. Accordingly, people’s mental health during pandemics and appropriate and timely strategies to reduce complications, especially for vulnerable groups.

## Data Availability

The dataset of the present study is available upon reasonable request. The corresponding author (Sajad Delavari) should be contacted if someone wishes to request the data from the survey (email: sajadd@gmail.com).

## Abbreviations

DAS: Depression, Anxiety, Stress
DASS: Depression, Anxiety, Stress Scale
BDI: Beck’s depression inventory
FSAQ: Four System Anxiety Questionnaire
Coef.: Coefficient
CI: Confidence Interval
NI: Not Inserted in the regression model

## Footnotes

1 Depression, Anxiety, Stress
